# Gamma oscillatory amplitude encodes stimulus intensity in primary somatosensory cortex

**DOI:** 10.3389/fnhum.2013.00362

**Published:** 2013-07-15

**Authors:** H. E. Rossiter, S. F. Worthen, C. Witton, S. D. Hall, P. L. Furlong

**Affiliations:** ^1^Aston Brain Centre, School of Life and Health Sciences, Aston UniversityBirmingham, UK; ^2^Sobell Department of Motor Neuroscience and Movement Disorders, UCL Institute of NeurologyLondon, UK; ^3^School of Psychology, Faculty of Science and Technology, Plymouth UniversityDrake circus, Plymouth, UK

**Keywords:** gamma, primary somatosensory cortex, pain, electrical, stimulus intensity

## Abstract

Gamma oscillations have previously been linked to pain perception and it has been hypothesized that they may have a potential role in encoding pain intensity. Stimulus response experiments have reported an increase in activity in the primary somatosensory cortex (SI) with increasing stimulus intensity, but the specific role of oscillatory dynamics in this change in activation remains unclear. In this study, Magnetoencephalography (MEG) was used to investigate the changes in cortical oscillations during four different intensities of a train of electrical stimuli to the right index finger, ranging from low sensation to strong pain. In those participants showing changes in evoked oscillatory gamma in SI during stimulation, the strength of the gamma power was found to increase with increasing stimulus intensity at both pain and sub-pain thresholds. These results suggest that evoked gamma oscillations in SI are not specific to pain but may have a role in encoding somatosensory stimulus intensity.

## Introduction

Primary somatosensory cortex (SI) is implicated in the processing of sensory-discriminative aspects of pain, such as stimulus intensity, location, and duration (Treede et al., 1999). In pain experiments, SI activation is generally observed in the hemisphere contralateral to the delivered stimulus (Ploner et al., 1999, 2000; Timmermann et al., 2001; Bornhovd et al., 2002). The strength of activation in SI has been found to increase correspondingly with increasing stimulus intensity in positron emission tomography (PET), functional magnetic resonance imaging (fMRI), and magnetoencephalography (MEG) (Coghill et al., 1999; Timmermann et al., 2001; Bornhovd et al., 2002; Della Penna et al., 2004). Bornhovd et al. (2002) used fMRI to examine the effects of laser stimuli at various intensities and observed that the amplitude of SI blood oxygen level dependent (BOLD) activation discriminated between non-painful trials, implicating its role in stimulus intensity encoding. Timmermann et al. (2001) found an increase in the amplitude of SI somatosensory evoked potential (SEP) responses using MEG, which exhibited an exponential relationship with stimulus intensity and the participant's pain ratings.

While PET and fMRI benefit from high spatial resolution, capable of resolving the source of neural activity with great accuracy, the temporal resolution of these techniques is unable to resolve the time course of activity at these loci. Conversely, electroencephalography (EEG) is able to distinguish changes in activity on a millisecond timescale, but is limited in its ability to provide accurate spatial localization of the source of these activities. However, advances in source level analysis approaches, such as beamforming methods like synthetic aperture magnetometry (SAM) (Van Veen et al., 1997; Vrba and Robinson, 2001), enable MEG to achieve higher spatial resolution (Hillebrand et al., 2005). The advantage of this approach is the ability to resolve the location of the neural generators and reconstruct the time course of power changes on a millisecond timescale. In particular, MEG affords the ability to determine the focal changes in neuronal network oscillatory activity in response to sensory stimuli in different regions of the cortex.

Neuronal network oscillations in the cortex, as measured with MEG, are emergent properties of phase synchronization between pyramidal cells. A number of cortical areas, including somatosensory cortex, exhibit spontaneously occurring oscillations in the mu frequency (~10 Hz), beta frequency (15–30 Hz), and gamma frequency (30–100 Hz) (Pfurtscheller and Lopes Da Silva, 1999; Roopun et al., 2006; Hall et al., 2010). Modulation of power in different frequency bands has been linked to particular states or tasks (Hari and Salmelin, 1997). Of particular interest is the gamma frequency, which has been proposed as a mechanism of temporal integration or “binding” of salient stimulus features across different sensory cortices (Engel and Singer, 2001). It has been observed that changes in gamma are more spatially discrete and somatotopically specific than lower frequency oscillations (Crone et al., 1998). A distinction must be made as to whether these oscillations are time-locked (evoked) to stimulus onset or not (induced) as this may reflect different physiological mechanisms (Tallon-Baudry and Bertrand, 1999). In the motor cortex, gamma oscillations are shown to be associated with the magnitude of output generated (Muthukumaraswamy, 2010). In the visual cortex, gamma shows a correspondence with the stimulus features such as spatial frequency, stimulus size and contrast, and this has been seen with evoked (Busch et al., 2004; Frund et al., 2007; Schadow et al., 2007) and induced (Adjamian et al., 2004b; Hall et al., 2005) gamma oscillations. Across the cortex, gamma has a proposed involvement in a number of cognitive tasks (Ward, 2003) and has been implicated as an attentional correlate in the processing of somatosensory stimuli in SI (Bauer et al., 2006; Hauck et al., 2007).

In studies exploring the processing of pain, an increase in gamma has been seen in SI in response to both painful electrical (Chen and Herrmann, 2001; De Pascalis et al., 2004; De Pascalis and Cacace, 2005; Hauck et al., 2007, 2008) and painful laser stimuli (Gross et al., 2007) as well as prefrontal regions (Croft et al., 2002). De Pascalis et al. (2004; De Pascalis and Cacace, 2005) observed “phase-ordered” (evoked) 40 Hz gamma oscillations, in central EEG electrodes in response to painful electrical stimuli. The amplitude of these gamma oscillations, which were attributed to SI, corresponded to the subjective pain ratings of the participants. These oscillations were diminished during hypnotic analgesia, suggesting that these gamma oscillations may be involved in higher-order top-down processing of pain. Previous studies have highlighted the importance of oscillatory dynamics in pain processing in somatosensory cortex (Worthen et al., 2011).

Hauck et al. (2007) observed induced gamma oscillations in response to painful intracutaneous electrical stimulation over the somatosensory cortices. An increase in oscillatory power was observed at two separate gamma frequencies, the higher oscillation being linked to attention to the stimulus. Importantly, some studies have attributed the increase in gamma power in SI to pain perception (Gross et al., 2007; Schulz et al., 2012; Zhang et al., 2012). Gross et al. (2007) reported that for stimuli of the same intensity, around pain threshold, the induced gamma amplitude was greater when participants rated the stimulus as painful compared to when they rated it as non-painful. Zhang et al. (2012) found that induced gamma oscillations predicted subjective pain intensity regardless of the saliency of the stimulus.

Other studies however, have observed increases in gamma power in SI following the delivery of non-painful somatosensory stimuli (Tecchio et al., 2003, 2008; Fukuda et al., 2008) Fukuda et al. (2008) found that the gamma oscillations seen in their study were initially phase-locked (evoked) but became non-phase locked (induced) with time. These studies would suggest that increases in gamma power in SI are not simply a signature of the delivery and processing of painful stimuli.

Previous studies, exploring the role of gamma in SI, have examined responses to either painful stimuli or non-painful stimuli, but few have explored both in the same experiment. This study aimed to investigate whether gamma oscillations in SI are a phenomenon specific to the perception and processing of painful stimuli. A source-level MEG approach was used, to examine the oscillatory signatures in SI using a train of somatosensory electrical stimuli at a range of non-painful and painful intensities.

## Materials and methods

### Participants

Twelve healthy participants (4 males; age range 24–43 years; 2 left-handed) took part in this study. All were free of any neurological or pain disorders and none were taking medication at the time of the study. Informed consent was obtained from all participants and the local ethics committee approved the experimental protocol.

### Stimuli

Electrical pulses generated by a constant current stimulator (Model: Digitimer Ltd, Welwyn Garden City, DS7A) were delivered to the right index finger of each participant via two pad electrodes positioned approximately 1 cm apart on the lateral surface of the digitus secundus, at the middle and proximal phalanx.

### Stimulus calibration

The experiment consisted of the delivery of four different stimulus intensities. These are referred to as: “low sensation,” “high sensation,” “low pain,” and “high pain” throughout. These stimulus intensities were determined immediately prior to the experiment using a staircasing procedure, by administering trains of electrical pulses at 7 Hz and increasing the current incrementally from 0 mA at a rate of ~0.5 mA/s. In our staircasing method, the participants were asked to verbally report when they could first feel the stimulus as the stimulus intensity was increased from 0 mA. Upon reported detection, the intensity was then reduced until the stimulus was no longer felt and then increased again until participants once again reported its presence. This was done twice to ensure accurate thresholds. The same method was used to assess pain threshold and pain tolerance. Participants used a visual analog scale (VAS) to report these thresholds ranging from 0 (no sensation) to 10 (worst pain imaginable). These points were: initial sensation (VAS = 1), pain threshold (VAS = 4), and pain tolerance (VAS = 7). Using these measurements, the four intensities were determined as: low sensation (25% between initial sensation and initial pain), high sensation (75% between initial sensation and initial pain), low pain (25% between initial pain and pain tolerance), and high pain (75% between initial and pain tolerance) using a similar method to that previously described by Hobson et al. (1998).

### Stimulus delivery

**Figure 1:**
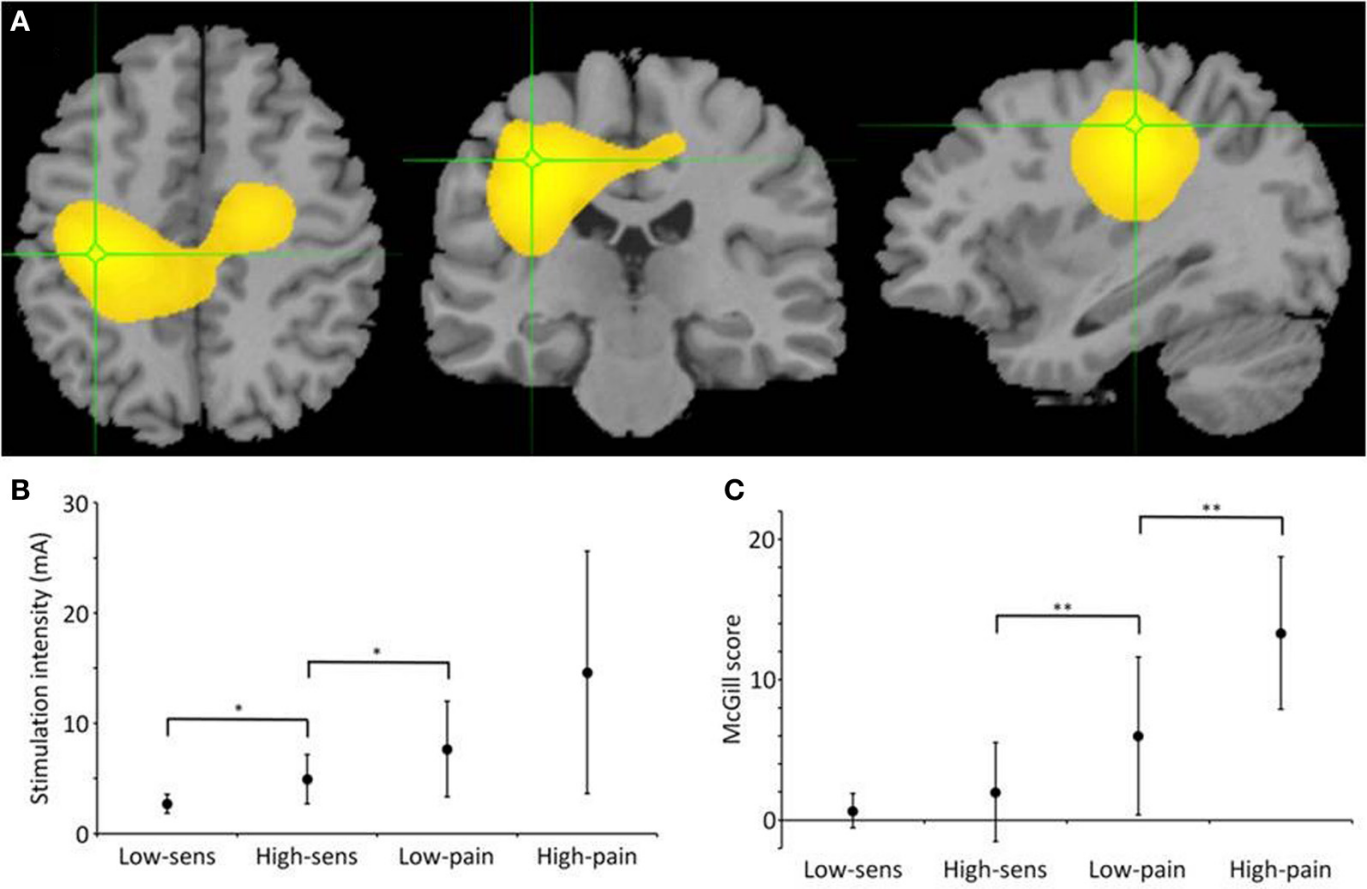
**Gamma source localization, stimulation and pain intensities. (A)** Average map of change in gamma across participants, located in post-central gyrus (Talairach coordinate = −33.1, −24.1, 45.0) **(B)** Graph showing actual stimulus intensity used at each level in mA. Significant differences between observations are shown on the graphs (^**^p < 0.001, ^*^p < 0.05). **(C)** Graph showing McGill score across the four different stimulus intensities, significant difference in scores between high sensation and low pain and also between low pain and high pain.

During the experiment, stimuli were delivered as trains of 200 μ s pulses, at a frequency of 7 Hz, with each train lasting for 2 s (14 stimuli per train). The experiment was arranged into four separate blocks, one for each stimulus intensity, lasting approximately 5 min in total. Each block consisted of 60 trials of 5 s. Each trial consisted of a 2 s train of stimulation, separated by 3 s of rest. The order in which the stimulus intensities were given was pseudo-randomized across participants. Participants were instructed to keep their eyes open and to focus on a central point, to minimize eye movement. In order to provide details of their subjective perception of the sensation and pain they received, each participant was instructed to fill out a McGill Pain Questionnaire (Melzack, 1975, 1987, 2005) after each block, the scores from the pain and sensory descriptors were summed and used as a rating for each stimulus intensity (Figure 1B).

### MEG recordings

Participants were seated in a magnetically shielded room for each of the 5 min recordings. Neural activity was recorded using a 275-channel CTF MEG system (CTF Systems Inc., Vancouver, Canada) at a sampling rate of 1200 Hz, with 3^rd^ order gradient noise reduction and DC-offset removal, based on the whole trial. The data were processed using a 50 Hz notch filter with a width of 0.6 Hz and a band-pass filter of 1–300 Hz. Each trial was manually examined to identify blink and muscle artifacts and the trial was removed if necessary. A 3-dimensional digitizer (Polhemus Isotrak, Kaiser Aerospace Inc., Colchester, Vermont, USA) was used to digitize the surface of the participants head and this information was then co-registered with the individual participant's anatomical MRI (Magnetom Trio, Siemens, Erlangen), using a comparable approach to that described previously, which gives a spatial accuracy ≤5 mm (Singh, 1995; Adjamian et al., 2004a).

### Data analysis

The SAM beamformer algorithm (Van Veen et al., 1997; Vrba and Robinson, 2001) was used to spatially localize the change in oscillatory power between active and passive periods. The oscillatory power difference between conditions is visualized as a t-statistic on the participants co-registered MRI; the details of this technique are described in detail by Barnes and Hillebrand (2003) and it has been successfully applied previously in sensory studies (Fawcett et al., 2004; Furlong et al., 2004; Hall et al., 2005). Here, SAM analyses were performed in the mu (6–9 Hz), beta (15–30 Hz), and gamma range (30–80 Hz) between the 2 s stimulation phase (active) and the 2 s pre-stimulation phase (passive) in the four different intensity blocks. The gamma band was further analysed around each electrical stimulation from 0–140 ms and the same window from the rest phase.

Coordinates from the anatomical MRI were determined from the peak changes in gamma power which were located in SI, from which “virtual electrodes” (VEs) were computed. In brief, these are a spatially discrete reconstruction of the neural activity at the location of interest, with the temporal resolution of the original recording (Barnes and Hillebrand, 2003). Using the VE from the high pain localization, time-frequency analysis of the envelope of stimulation was then performed for each stimulation intensity. Specifically, a bootstrapping approach was used to determine the percentage change in power across the frequency range (0–100 Hz) between each 2 s stimulation period and the 2 s pre-stimulation baseline (Graimann et al., 2002) and a Morlet-wavelet transform was used to create a time-frequency spectrogram of each stimulation intensity. Normalized group spectrograms for each stimulation intensity were then created from the combined data of those participants who showed changes in the gamma band, to visualize the oscillatory activity across the group.

In order to differentiate the evoked and induced components of the response, three sets of spectrograms were computed. The average of spectrograms for each epoch within a condition showed both induced and evoked changes in spectral power for that condition. Evoked activity alone was estimated using a spectrogram of the averaged time series for each condition, excluding any induced power changes not time-locked to the stimulus. Finally, to estimate the activity which was solely induced, the average time series for a given condition was subtracted from each epoch within that condition, and the spectrograms of the resulting time series were averaged (Hauck et al., 2007).

The dependence of the change in the power of the broad gamma band upon stimulus intensity was tested using a one-way repeated measures ANOVA to calculate the interaction between the averaged gamma power increase (30–80 Hz) over the 2 s stimulation period and the stimulation intensity.

## Results

### SAM localisation of gamma

The SAM analysis revealed a significant change in gamma frequency (30–80 Hz) in left SI, contralateral to the stimulated right index finger, in the 12 participants when analysing around each electrical pulse (Figure 1A). Based upon the presence of a reliable gamma peak in SI, the data were then further analysed to determine the relationship between stimulation intensity, pain rating and gamma power in the virtual electrode at the peak of the gamma power.

### Stimulation intensity and pain ratings

The stimulation intensities determined at the calibration stage, were significantly different (*t* = 3.81, *p* = 0.013) between the low sensation (mean = 2.70, *SD* = 0.85 mA) and high sensation (mean = 4.93, *SD* = 2.23 mA) and a significant difference (*t* = 2.65, *p* = 0.045) between the high sensation and low pain (mean = 7.65, *SD* = 4.33 mA). However, as a consequence of the variance across participants, there was no significant difference (*t* = 1.94, *p* = 0.11) between low pain and high pain (mean = 14.6, *SD* = 10.99 mA) (Figure 1B).

Conversely, the McGill scores for each stimulus intensity rating, revealed no significant difference (*t* = 0.97, *p* = 0.38) between the low sensation (mean = 0.67, *SD* = 1.2) and high sensation (mean = 2.0, *SD* = 3.5). However, it confirmed a significant difference (*t* = 4.11, *p* = 0.009) between high sensation and low pain (mean = 6.0, *SD* = 6.5) and a significant difference (*t* = 4.25, *p* = 0.008) between low pain and high pain (mean = 13.3, 5.42) (Figure 1C).

Changes in mu and beta frequency bands were also seen in a number of participants. A decrease in beta power was seen across the 2 s stimulus train in 9 out of 12 participants. An increase in mu power was clear in 4 participants although this was around the same frequency as that of the electrical stimulation. A one-way repeated measures ANOVA of both mu and beta power did not show a significant relationship with stimulus intensity [mu: *F*_(3)_ = 0.88, *p* = 0.48, beta: *F*_(8)_ = 1.58, *p* = 0.21].

### Gamma power and stimulation intensity

**Figure 2:**
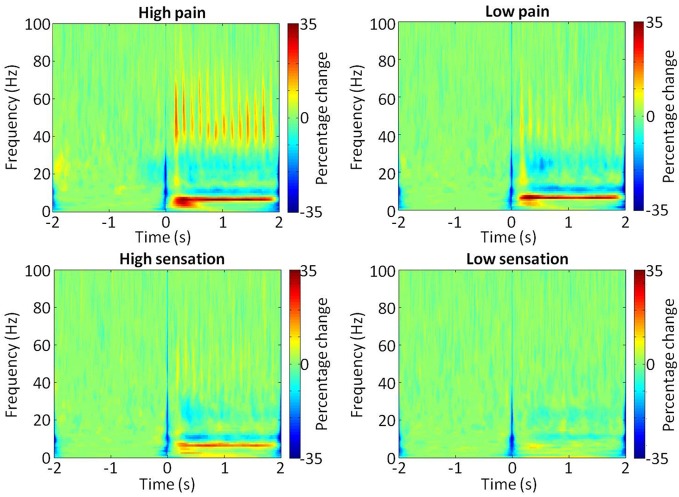
**Time-frequency representation of SI during stimulation.** Normalized group average bootstrap spectrograms showing changes in power (%) at each frequency (0–100 Hz) during the stimulation train (0–2 s). Each of the four stimulus intensities are shown, with the color scale representing percentage change from baseline (2 s preceding stimulus onset).

**Figure 3:**
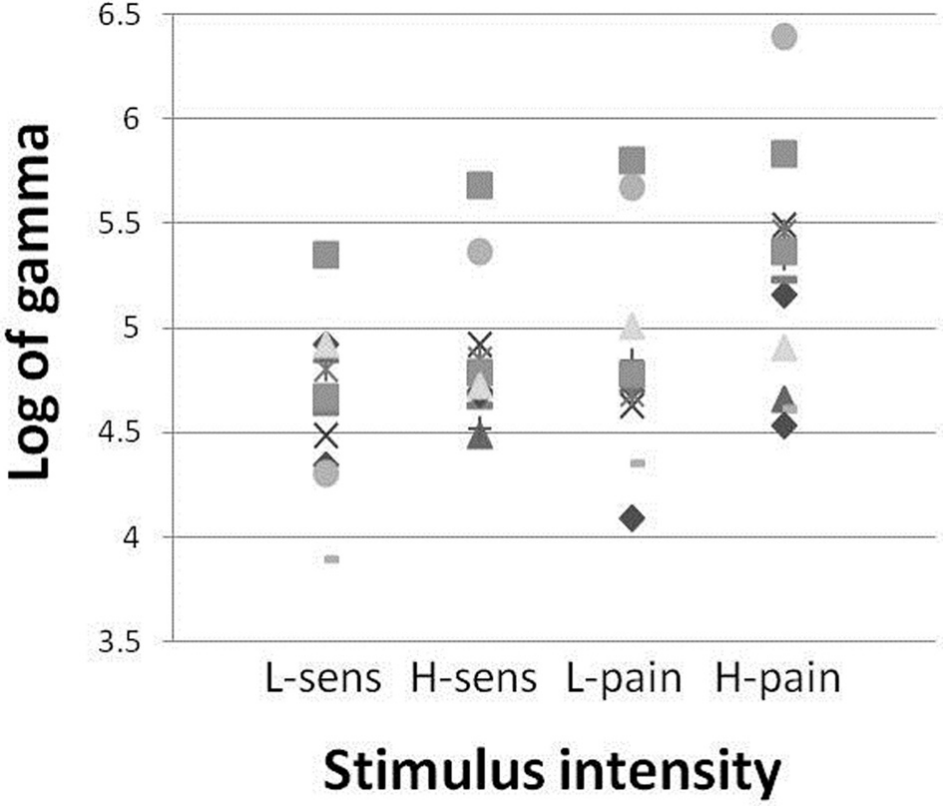
**Gamma amplitude vs. stimulation intensity.** Gamma (30–80 Hz) power increase (log of area under curve of gamma power spectra) with each symbol representing one participant, at each of the stimulation intensities.

The area under the curve of the power spectra in the gamma range was calculated over the 2 s stimulus period and then the log of this value was taken to provide a measure of gamma power. Analysis of the increase in gamma (30–100 Hz) power during each of the stimulation intensities, using a one-way repeated measures ANOVA of gamma power × stimulation intensity, confirmed a general dependence of gamma amplitude on stimulation intensity [*F*_(11)_ = 6.07, *p* = 0.002]. Time-frequency spectrograms (Figure 2) demonstrated a clear increase in the >30 Hz gamma power range during stimulation, in the high sensation, low pain and high pain intensities, which was not apparent in low sensation. The gamma power increase appeared to follow the periodicity of the stimulation train at 7 Hz, consistent with the observation of a 7 Hz increase in the spectrogram. In addition, decreases were observed in the mu (~10 Hz) and beta (15–30 Hz) frequency ranges, consistent with effects observed in previous stimulation studies (Cheyne et al., 2003; Gaetz and Cheyne, 2006; Ploner et al., 2006). Analysis of the mean gamma power (30–100 Hz) confirms an increase with stimulus intensity (Figure 3). Furthermore, analysis performed in order to explore phase-locking revealed that the gamma oscillations seen in this study were evoked.

## Discussion

The results of this study suggest that the amplitude of the evoked gamma response in SI shows a direct relationship with the intensity of the stimulus, rather than showing a specific dependence upon pain. An increase in evoked gamma oscillatory power was observed in response to both painful and non-painful stimulus trains and there was no obvious change in the temporal pattern between painful and non-painful stimulation, with both appearing to follow the time-course of the stimulus train. The appearance of this evoked gamma increase and temporal structure, suggests that the SI response in the gamma frequency range is encoding the arrival of somatosensory stimuli rather than a pain stimulus per se. Importantly, the results here demonstrate a relationship between the change in the individual stimulus intensity and the change in gamma amplitude response. Indeed, there was no clear difference in the increase in the gamma amplitude in response to high sensation and low pain stimuli. This implies that the perceptual difference between these two conditions, specifically the presence of pain, was not accompanied by a difference in the gamma amplitude.

These results are consistent with previous human studies on SI function, which suggest that it plays a central role in the sensory-discriminative properties of somatic stimuli (Coghill et al., 1999; Treede et al., 1999; Timmermann et al., 2001; Bornhovd et al., 2002; Della Penna et al., 2004; Worthen et al., 2011). This is further consistent with primate experiments, in which the discharge frequency of SI neurons was shown to increase in response to increasing noxious thermal stimulation (Kenshalo et al., 2000).

The gamma oscillations observed in this study were in a similar frequency range to those seen by Hauck et al. (2007) and Gross et al. (2007), although these gamma oscillations were reported to be induced rather than evoked. It is possible that while evoked gamma oscillations appear to play a role in sensory-discriminative aspects of somatic stimuli, induced gamma oscillations may have a more high-level cognitive role in sensory and pain processing involving attention and perception (Tallon-Baudry and Bertrand, 1999). Fukuda et al. (2008) saw gamma oscillations in response to sensory stimuli that were evoked to begin with but then became induced over time, perhaps in our study as we have a train of pulses we are only capturing the evoked component of these gamma oscillations.

The question of specificity of the amplitude of both the gamma power and evoked potential amplitude, has been explored using EEG to record cortical responses to repetitive laser stimuli (Iannetti et al., 2008; Mouraux and Iannetti, 2009; Iannetti and Mouraux, 2010). Iannetti et al. (2008) demonstrated that evoked potential amplitude is dependent upon stimulus saliency and attention. A recent study by Zhang et al. (2012) demonstrated that the amplitude of induced gamma-band oscillations in response to painful stimuli is predicted by the subjective pain intensity, irrespective of the saliency of the stimulus. The findings in our study are consistent with these observations, in so far as they demonstrate greater amplitude in the high pain compared to low pain conditions. However, we suggest that the evoked gamma oscillations found in our study are not predicated upon the perception of pain and that the increase in gamma power between intensities of painful stimuli was not separable from an increase that was dependent upon stimulus intensity. Importantly, our observation of an increase in evoked gamma with non-painful stimuli, is consistent with observations in other studies using non-painful vibrotactile and touch pulse stimuli (Ross et al., 2013). Furthermore, the observation of increases in gamma activity from non-painful somatosensory stimuli are consistent with the observation of increased SI gamma power (25–70 Hz) following stimulation of the forepaw in an anaesthetized rat (Sumiyoshi et al., 2012). This is of particular interest, as the same study showed that the neurovascular coupling of the fMRI signal in SI was mainly driven by the gamma response (Sumiyoshi et al., 2012). It was not clear from these studies whether the gamma oscillations seen were evoked or induced. Our results are further consistent with stimulation studies that show that the stimulation of SI cortex using transcranial alternating current stimulation (tACS) at gamma frequency (52–70 Hz) generates a tactile sensation in the contralateral hand (Feurra et al., 2011). The generation of sensation rather than pain in the associated area, implies that the gamma signal in this range encodes stimulus intensity rather than pain perception.

The findings in the gamma frequency range are supported by the observation of amplitude changes in the mu and beta frequency ranges, which show a change in power in response to somatosensory stimuli. An increase in mu was seen in 4 of the 12 participants around 6–9 Hz, however this could reflect the frequency of the electrical stimulation which was 7 Hz. In keeping with previous reports, the changes in these frequency ranges were independent of pain perception and occurred in response to non-painful stimulation (Cheyne et al., 2003; Ploner et al., 2006).

Gamma oscillations have been linked to attentional processing in response to both tactile (Bauer et al., 2006) and painful (Hauck et al., 2007; Tiemann et al., 2010) stimuli. In our study we are unable to separate out attentional effects, though an increase in attention with higher pain intensity would be predicted.

In summary, the results of this study suggest that evoked gamma oscillations may offer a possible mechanism for SI to encode stimulus intensity regardless of participant perception of pain.

### Conflict of interest statement

The authors declare that the research was conducted in the absence of any commercial or financial relationships that could be construed as a potential conflict of interest.
